# Supplementary material for: Predicting the Young’s Modulus of Silicate Glasses using High-Throughput Molecular Dynamics Simulations and Machine Learning
Source: Sci Rep. 2019 Jun 19;9:8739. doi: 10.1038/s41598-019-45344-3 (PMC6584533; doi:10.1038/s41598-019-45344-3)
Supplement: Supplementary file 1 — Supplementary Information [file 41598_2019_45344_MOESM1_ESM.pdf]

Supplementary Information

Predicting the Young's Modulus of Silicate Glasses using High-Throughput Molecular Dynamics Simulations and Machine Learning

Kai Yang[1], Xinyi Xu[1], Benjamin Yang[1], Brian Cook[1], Herbert Ramos[1], N.M. Anoop Krishnan[2,3], Morten M. Smedskjaer[4], Christian Hoover[5], and Mathieu Bauchy[1,*]

[1]Physics of AmoRphous and Inorganic Solids Laboratory (PARISlab), University of California, Los Angeles, CA 90095, U.S.A.

[2]Department of Civil Engineering, Indian Institute of Technology Delhi, Hauz Khas, New Delhi, India 110016

[3]Department of Material Science and Engineering, Indian Institut e of Technology Delhi, Hauz Khas, New Delhi, India 110016

[4]Department of Chemistry and Bioscience, Aalborg University, 9220 Aalborg, Denmark

[5]School of Sustainable Engineering and the Built Environment, Arizona State University, Tempe, AZ 85287, U.S.A

[*]Corresponding author: Prof. Mathieu Bauchy, bauchy@ucla.edu

Machine learning model based on experimental data only

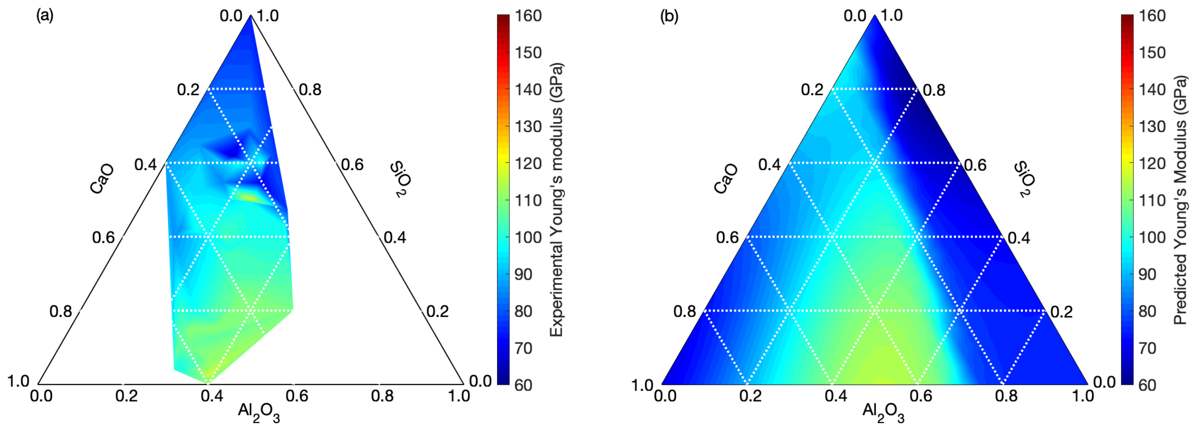

Figure S1. (a) Ternary diagram showing the Young's modulus values from 109 experiments as a function of composition in the CaO–Al_2O_3–SiO_2 glass system. **(b)** Ternary diagram showing the Young's modulus values predicted by a back-propagation ANN model with 1 hidden layer and 5 neurons trained based on the experimental data presented in (a).

Overfitting and underfitting

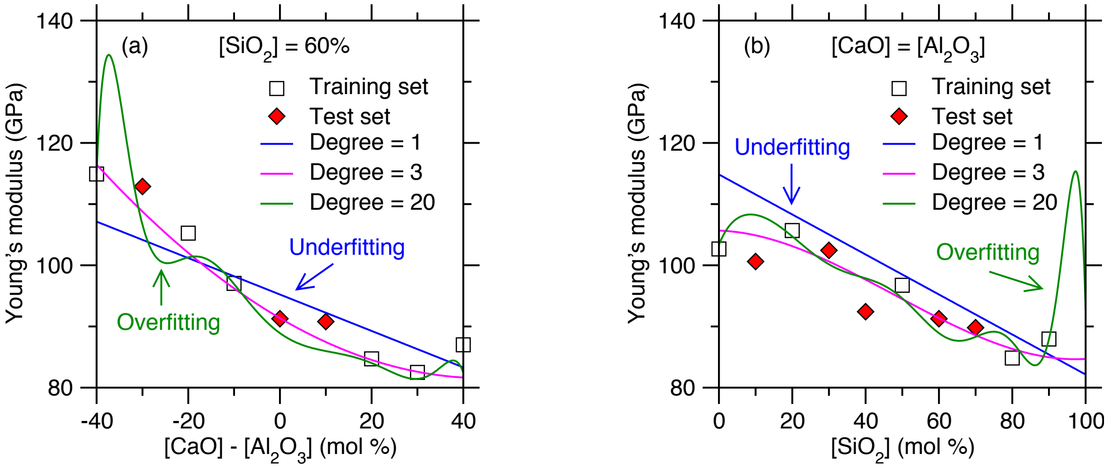

Figure S2. Comparison between the Young's modulus values computed by molecular dynamics simulations (wherein the training and test sets are indicated as white and red symbols, respectively) and predicted by select polynomial regression models with polynomial degrees of 1 (underfitted), 3 (optimal), and 20 (overfitted) for the series of compositions **(a)** $(CaO)_x(Al_2O_3)_{40-x}(SiO_2)_{60}$ and **(b)** $(CaO)_x(Al_2O_3)_x(SiO_2)_{100-2x}$.

We investigate the manifestations of underfitting and overfitting in ML-based modelling. To this end, we focus on the example of PR—since the other methods considered herein do not yield any clear signature of overfitting. To this end, Fig. S2 shows the Young's modulus values predicted by select PR models trained with varying polynomial degrees, namely, 1 (underfitted), 3 (optimal degree), and 20 (overfitted). Overall, we observe that the underfitted model (linear model with degree = 1) is obviously too simple to properly capture the non-linear nature of the dataset. In contrast, due to its high complexity, the overfitted model is able to memorize the "noise" of the simulation data used in the training set. By encoding such noise in high-degree polynomes, the overfitted model offers a poor prediction of the test set. Specifically, overfitting results in the appearance of some intense spurious ripples toward the edges of the compositional domain. Overall, the optimal model (i.e., degree 3) offers the best ability to capture the non-linearity of the data while filtering out the noise of the simulation data. These results illustrate the requirement of properly tuning the level of complexity of ML models.

Machine learning algorithms. We now detail the different learning methods used herein. We first focus on the polynomial regression (PR) method[1]. PR is a special case of multiple linear regression that includes higher degree polynomial terms and treats these higher degree polynomes as other independent variables. In general, the Nth degree PR method can be described as:

$$Y = \beta_0 + \sum_{i=1}^{N} \beta_i X^i \quad (4)$$

where X is the input, Y is the output, and the β_i terms are the fitting parameters corresponding to each degree i. Here, we adopt the multivariate polynomial regression with two independent variables, which can be expressed as:

$$Y = \beta_0 + \sum_{i=1}^{N} \beta_i X_1^i + \sum_{j=1}^{N} \beta_j X_2^j + \sum_{k=1}^{N-1} \beta_k X_1^k X_2^{N-k} \tag{5}$$

where X_1 and X_2 are the two input variables (i.e., the composition terms *x* and *y* herein). The least-square method is then used to identify the coefficients β_i that minimize the sum of squared difference between the "real" stiffness values (i.e., computed by MD) and those predicted by the PR method (i.e., *Y*) during the training phase. The complexity of PR models depends on the choice of the Nth polynomial degree considered during training.

One of the major disadvantages of the least-squares approximation is that it tends to overfit the training data. LASSO (least absolute shrinkage and selection operator) regression offers a useful solution to decrease the complexity of the model and, thereby, limit the risk of overfitting[2]. This is accomplished by starting with the cost function used in PR (i.e., the sum of squared difference between "real" and "predicted" values) and adding an additional term that further penalizes complex models. The new cost function that needs to be minimized is then defined as:

$$\sum_{i=1}^{N} \left(Y_i - \beta_0 - \sum_{j=1}^{p} X_{ij} \beta_j \right)^2 + \lambda \sum_{j=1}^{p} |\beta_j| \tag{6}$$

where λ is a hyperparameter that is used to control the weight of the penalty associated with the complexity of the model. In practice, LASSO will force some of the β_j coefficients to be zero to minimize the value of the cost function, which results in a decrease in the complexity of the model. The degree of complexity of LASSO models can be tuned by adjusting the value of λ, namely, increasing values of λ yield simpler models.

The random forest (RF) method relies on the creation of a "forest," that is, an ensemble of decision trees[3]. The RF approach builds a tree by randomly choosing *n* samples from the training set (bootstrap method). Then, at each node, it uses a randomly selected subset of variables to choose the best split to construct trees. Random forest runs input data on all n_t trees and yields a prediction that is the average of all values returned by each tree:

$$Y(X) = \frac{1}{n_t} \sum_{i=1}^{n_t} Y_i(X) \tag{7}$$

where $Y_i(X)$ is the individual value predicted by one of the trees for an input vector X and $Y(X)$ is the overall output of the random forest model with n_t trees[4]. The degree of complexity of RF models are characterized by the number of variables in the random subset and trees in the forest[5].

Artificial neural networks (ANN) aim to mimic the learning process of human brains. ANN models consist of an input layer that is connected to an output layer via some "hidden" layers of neurons. Each neuron takes as inputs the signals from the previous layer and produces a new output (to be used as input by the neurons from the next layer). The output Y_i of a neuron i in one of the hidden layers is calculated as:

$$Y_i = s(\sum_{i=1}^{N} w_i X_i + T_i^{\mathrm{hid}}) \tag{8}$$

where $s()$ is an activation function, N is the number of input neurons in the previous layer, X_i are the input values, w_i are the weight associated with each edge of the network, and T_i^{hid} is the threshold term of hidden neurons[4]. To capture the non-linearity in the relationship between composition and stiffness data, we adopt herein a sigmoid function as activation function:

$$s(u) = \frac{1}{1+ e^{-u}} \tag{9}$$

We adopt here the resilient backpropagation (BP) algorithm to train the neural network model, which allows the network to learn from its errors[6]. The BP algorithm is an efficient method as it allows one to adjust the weight w_i by calculating the gradient of loss function E_{loss}. To measure the error between the predicted and real outputs after a training sample has propagated through the network, we use the square of Euclidean distance to calculate the loss function E_{loss} over n training outputs as:

$$E_{\mathrm{loss}} = \frac{1}{2n} \sum_{i=1}^{n} \left\| (Y_i - Y_i^{'}) \right\|^2 \tag{10}$$

where Y_i are the predicted outputs and Y_i' is the read values (i.e., the simulated Young's modulus values). Then, after k iterations, each weight w_i is modified by applying an increment:

$$w_i^{(k+1)} = w_i^{(k)} + \Delta^{(k)} w_i \tag{11}$$

where $w_i^{(k+1)}$ is the updated weight, $w_i^{(k)}$ is the weight before update, and $\Delta^{(k)} w_i$ is the increment. The latter is calculated by following the steepest decreasing gradient in the error function as:

$$\Delta^{(k)} w_i = -\gamma_i^{(k)} \mathrm{sgn}(\nabla_i E^{(k)}) \tag{12}$$

where $\mathrm{sgn}()$ denotes the "sign function", $\nabla_i E^{(k)}$ denotes the partial derivative (i.e., gradient) of the error function $E^{(k)}$ with respect to weight w_i at the kth iteration, and $\gamma_i^{(k)}$ is the learning rate at k^{th} iteration. The model is iteratively refined until all the absolute values of partial derivatives of the error function become smaller than a threshold value.

References

1. Stigler, S. M. Optimal Experimental Design for Polynomial Regression. *J. Am. Stat. Assoc.* **66**, 311–318 (1971).
2. Tibshirani, R. Regression Shrinkage and Selection via the Lasso. *J. R. Stat. Soc. Ser. B Methodol.* **58**, 267–288 (1996).
3. Breiman, L. Random Forests. *Mach. Learn.* **45**, 5–32 (2001).
4. Anoop Krishnan, N. M. *et al.* Predicting the dissolution kinetics of silicate glasses using machine learning. *J. Non-Cryst. Solids* **487**, 37–45 (2018).
5. Liaw, A. & Wiener, M. Classification and Regression by RandomForest. *Forest* **23**, (2001).
6. Rumelhart, D. E., Hinton, G. E. & Williams, R. J. Learning representations by back-propagating errors. *Nature* **323**, 533–536 (1986).
